# Supplementary material for: What are the odds? Poor compliance with UK loot box probability disclosure industry self-regulation
Source: PLoS One. 2023 Sep 27;18(9):e0286681. doi: 10.1371/journal.pone.0286681 (PMC10530011; doi:10.1371/journal.pone.0286681)
Supplement: Attachment — Submitted filename: Revision Letter.docx [file pone.0286681.s001.docx]

Dear Prof Grima,

Thank you very much for this opportunity to submit a revised version of this manuscript. Reviewer comments have been included below in full in *italics*. Our point-by-point response is in purple and any relevant newly added text is in blue.

Yours sincerely,

Leon Y. Xiao

*Reviewer #1:*

*My sincere thanks to the editor for the opportunity to review this manuscript.*

*It has been a pleasure for me to read the manuscript which I believe makes a relevant contribution.*

*There are many virtues of the manuscript, starting with a topic that is highly current, that changes continuously and that has a profound social relevance. In addition, the authors who carried it out the research are highly recognized and well versed in the subject they address. The paper updates and builds on previous knowledge of the subject, providing current data that can inform different social agents who are concerned (and should be concerned) about this phenomenon.*

*I simply have a few issues that I think can be improved, but having dealt with them I believe that the paper will be useful for the readers of the journal and that it will make a scientific contribution to the research community.*

Thanks very much to Reviewer 1 for taking the time to read and comment on this manuscript.

*Here are a few minor issues that I have detected:*

*‘attempting to ban loot 82 boxes may also be impractical, as demonstrated by how Belgium failed to do so’ seems like a very rigorous statement…perhaps something more subtle as has not been able to tackle or be innefective would be more sensitive*

We have changed ‘as demonstrated by how Belgium failed to do so’ to ‘as demonstrated by Belgium’s ineffective attempt to do so’.

*I cannot help but wonder why is the manuscript so heavily focused on Iphone leaving out android. A quick search reveals that according to statista as ‘of June 2022, iOS had 50.99 percent of the market share of mobile operating systems in the United Kingdom (UK), while Android followed closely with over 48 percent’ Given that Android phones in general are cheaper that IPhone I wonder if a SES bias is not in place when only checking Iphones.*

*Moreover I think (I am not an expert on these matters) the work flow in the Play Story (from android) is different form that of the Apple Store, which could lead to very different results in Android phones. Perphas this should be addressed in the limitation section of the paper.*

This is a great point. We focused on the iPhone platform because the Chinese study that we wanted to compare our results to only studied that platform. We do intend to conduct a replication of this study on Android in the coming months. We have added the following to the limitation section as suggested by Reviewer 1:

In particular, a future study should consider the compliance situation on the Google Play Store for Android (see 60): this is because Apple iOS devices are generally more expensive than Android devices meaning that the two platforms might be used by different players and companies, such that the present iOS-based results may have been affected by socioeconomic status bias and therefore not replicate to the Android platform.

*Figure 2 is not very clear in my opinion, could it be made more clear of be explained better in text? (I found this information later in the manuscript on line 377) perhaps the Figure could be place closer to this information)*

We have added additional information about how the button was not sign-posted at where we first discuss this particular disclosure shown in Figure 2.

*On page 11 a sentence start at line 320 and end at line 327. Such a long sentence is very hard to follow.*

We have broken this one sentence into three separate sentences.

*How I would know which are the games that were analysed? How would I know which are games 1 and 26 (page 11, line 320) Could not they be presented in the manuscript as a table or igure?*

A list of all games studied and their numbering have been added to the manuscript as Table 1 as suggested by Reviewer 1. We have also gone through the manuscript to ensure that we mentioned the game’s title at all instances where a game’s numbering was mentioned.

*Why is the section labeled as ‘Confirmatory Analyses’ as confirmatory analysis. Could not be the title a little bit more informative? What is that the authors are trying to confirm, what were the analyses performed? I think that the section titles should be more informative overall*

*I found odd that the Discussion starts with Hypothesis 2 rather than Hypothesis 1. Even if this confirmed I think that it would be a much more natural way to start the discussion section.*

We have provided more detail after ‘Confirmatory Analyses’ to explain that this was for ‘Comparing Disclosure and Prevalence Rates.’ This was intended to differentiate preregistered confirmatory analyses with exploratory analyses included later in the Results section.

We began the discussion with Hypothesis 2 rather than Hypothesis 1 because Hypothesis 2 dealt with the broader question of how prevalent loot boxes are. We believe this is the better way to tell the story. We have decided not to switch the ordering of Hypotheses 1 and 2 because we preregistered them as such and are concerned that we might confuse readers who decide to read both the preregistration and the paper if we switched the ordering.

***Reviewer #2:***

*What are the odds? Poor compliance with UK loot box probability disclosure industry self-regulation*

We are grateful to Reviewer 2 for the time spent on reviewing this manuscript and for providing comments.

*Title can be reframed like:*

*What are the odds? UK loot box probability disclosure sector self-regulation noncompliance.*

We believe our title best represents the main idea of the paper. Considering also that Reviewer 1 has not raised any concerns about the title, we have decided not to change it.

*Abstract: I completely dissatisfied the overall abstract. I didn’t find much information on Purpose, need of the study, Methodology, findings and Practical Implications.*

We believe our abstract appropriately situates the study and presents the results. Considering also that Reviewer 1 has not raised any concerns about the abstract, we have decided not to change it.

*Introduction is less and not up to the mark. I didn’t find much information in the introduction section that can convince the readers that your research subject is interesting and that they should continue reading your paper. A detailed background is missing.*

*Literature review: It is quite dissatisfactory. Please focus on the research gap in the introduction. Strengthen the argument of your study by referring to a larger body of scientific literature, and clearly outline what is missing in the literature and what gap you wish to fill in.*

We believe our introduction section is sufficient. We have clearly set out where the gaps in the existing literature lie. We do not believe an extensive literature review is needed in this paper. Considering also that Reviewer 1 and reviewers at other venues where this paper was previously reviewed have not raised any concerns about the introduction section, we have decided not to change it.

*Please rewrite the whole Research methodology*

We believe our method section was written appropriately by detailing what we did. Considering also that Reviewer 1 and reviewers at other venues where this paper was previously reviewed have not raised any concerns about the method section, we have decided not to change it.

*The author has written a small portion of analysis and discussion part in conclusion.*

*There is a need of division of discussion and conclusion into two separate sections, with the conclusion section simply needing to give the most critical information in points so that readers can find what they want from the publication in a short time. The discussion section might need to add more subheadings, such as discussion of the findings, main contributions, policy recommendations, limitations, and future work of this study*

We believe our conclusion section presents the most critical information for readers. Considering also that Reviewer 1 has not raised any concerns about the conclusion section, we have decided not to change it.

We have included subheadings in the discussion section to sign-post readers about the immediately following paragraphs.
